# Lipid nanoparticles for delivery of RNA therapeutics: Current status and the role of *in vivo* imaging

**DOI:** 10.7150/thno.77259

**Published:** 2022-10-24

**Authors:** Han Na Jung, Seok-Yong Lee, Somin Lee, Hyewon Youn, Hyung-Jun Im

**Affiliations:** 1 Department of Applied Bioengineering, Graduate School of Convergence Science and Technology, Seoul National University, Seoul 08826, Republic of Korea.; 2 Department of Biomedical Sciences, Seoul National University Graduate School, Seoul 03080, Republic of Korea.; 3 Department of Molecular Medicine and Biopharmaceutical Sciences, Graduate School of Convergence Science and Technology, Seoul National University, Seoul 08826, Republic of Korea.; 4 Cancer Research Institute, Seoul National University College of Medicine, Seoul 03080, Republic of Korea.; 5 Department of Nuclear Medicine, Seoul National University Hospital, Seoul 03080, Republic of Korea.; 6 Research Institute for Convergence Science, Seoul National University, Seoul, 08826, Republic of Korea.

**Keywords:** Lipid nanoparticles, RNA therapeutics, Drug delivery system, Vaccines, *In vivo* imaging

## Abstract

Lipid nanoparticles (LNPs) have been one of the most successful nano-delivery vehicles that enable efficient delivery of cytotoxic chemotherapy agents, antibiotics, and nucleic acid therapeutics. During the coronavirus disease (COVID-19) pandemic, LNP-based COVID-19 messenger RNA (mRNA) vaccines from Pfizer/BioNTech and Moderna have been successfully developed, resulting in global sales of $37 billion and $17.7 billion, respectively, in 2021. Based on this success, the development of multiple LNP-based RNA therapeutics is gaining momentum due to its potential in vaccines and therapeutics for various genetic diseases and cancers. Furthermore, imaging techniques can be utilized to evaluate the pharmacokinetics and pharmacodynamics (PK/PD) effects, which helps target discovery and accelerates the development of LNP-based mRNA therapies. A thorough introduction and explanation of the components of LNPs and its functions along with various production methods of formulating LNPs are provided in this review. Furthermore, recent advances in LNP-based RNA therapeutics in clinics and clinical trials are explored. Additionally, the evaluation of PK/PD of LNPs for RNA delivery and the current and potential roles in developing LNP-based mRNA pharmaceutics through imaging techniques will be discussed.

## Introduction

RNA therapeutics have shown potential in various medical applications, including virus vaccines, cancer immunotherapy, and gene editing 1. The various drug delivery vehicles including lipid nanoparticles (LNP) for RNA therapeutics have been developed because of the instability of RNA. In 1978, the delivery of mRNA using liposomes was first reported 2. To further improve the encapsulation of the charged mRNA, cationic lipid based LNPs 3 and ionizable lipid based LNPs 4 were sequentially developed. Currently, ionizable lipids are considered as crucial components of LNP-based RNA therapeutics because they are positively charged at a low pH to enhance the encapsulation of negatively charged RNA, and the charge becomes less positive or almost neutral at physiological pH (~7.4), to reduce the toxicity 5. In addition, various mRNA engineering methods have been developed to enhance the stability and the translation efficacy of mRNA therapeutics, such as a selection of untranslated regions (UTRs), addition of a poly-A tail, capping, and nucleoside modification 6. Based on these advanced techniques, two LNP-based mRNA vaccines (BNT 162b2, Pfizer-BioNTech; and mRNA-1273, Moderna) were successfully developed and obtained authorization from regulatory agencies in 2020 in multiple countries 7. Additionally, multiple types of LNP-based RNA therapeutics are under active investigation to treat various infectious diseases and cancers 8.

Bioimaging has a crucial role in identifying drug targets and evaluating off-target effects and pharmacokinetics/pharmacodynamics (PK/PD) profiles of drugs 9, 10. Nanoparticles are considered as a suitable platform to utilize bioimaging because it is easy to incorporate various types of imaging contrasts in the nanoparticles which do not significantly affect their PK characteristics 11. Furthermore, the development of bioimaging techniques for therapeutics can result in the development of theranostic agents. The theranostic approach has drawn attention to the development of drugs due to the following advantages: 1) In the preclinical stage, it can help to select appropriate drug candidates based on the imaging results; 2) in clinical trials, companion imaging methods can be used to select patients for enrollment in clinical trials; 3) finally, in clinical practice, unnecessary treatment can be avoided by using companion imaging biomarkers for patient selection. With these benefits, theranostic agents have been successfully developed in recent years. For example, the U.S. Food and Drug Administration (FDA) approved ^177^Lu-dotatate (Lutathera) in neuroendocrine tumors and ^177^Lu-PSMA-617 (Pluctivo) in prostate cancer 12. Additionally, numerous studies have been conducted to develop theranostic nanoparticles because 1) nanoparticles can be functionalized with therapeutics and diagnostics easily and 2) various methods for controlled release of the therapeutics can be applied 13, 14.

**Figure 1:**
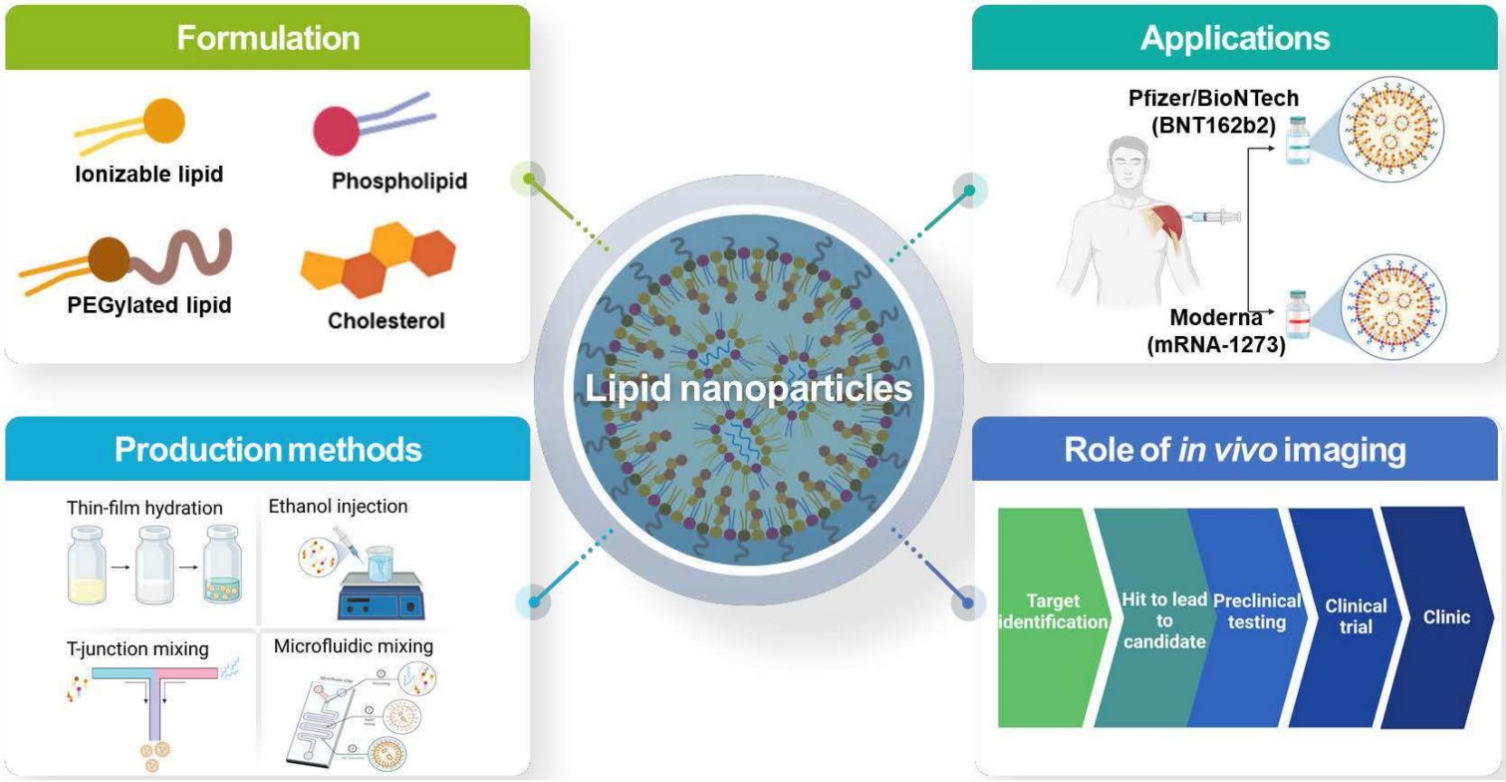
Overall schematic illustration of lipid nanoparticles for delivery of RNA therapeutics.

This review presents a comprehensive description of the currently available LNP production methods for LNP-based RNA therapeutics and LNP-based RNA therapeutics in clinics and clinical trials, and the PK/PD characteristics of LNP-based RNA therapeutics and the current and potential roles of imaging techniques in the development of LNP-based RNA therapeutics as shown in **Figure 1**.

## Development of LNP for RNA Delivery

### Lipid nanoparticle compositions

**Figure 2:**
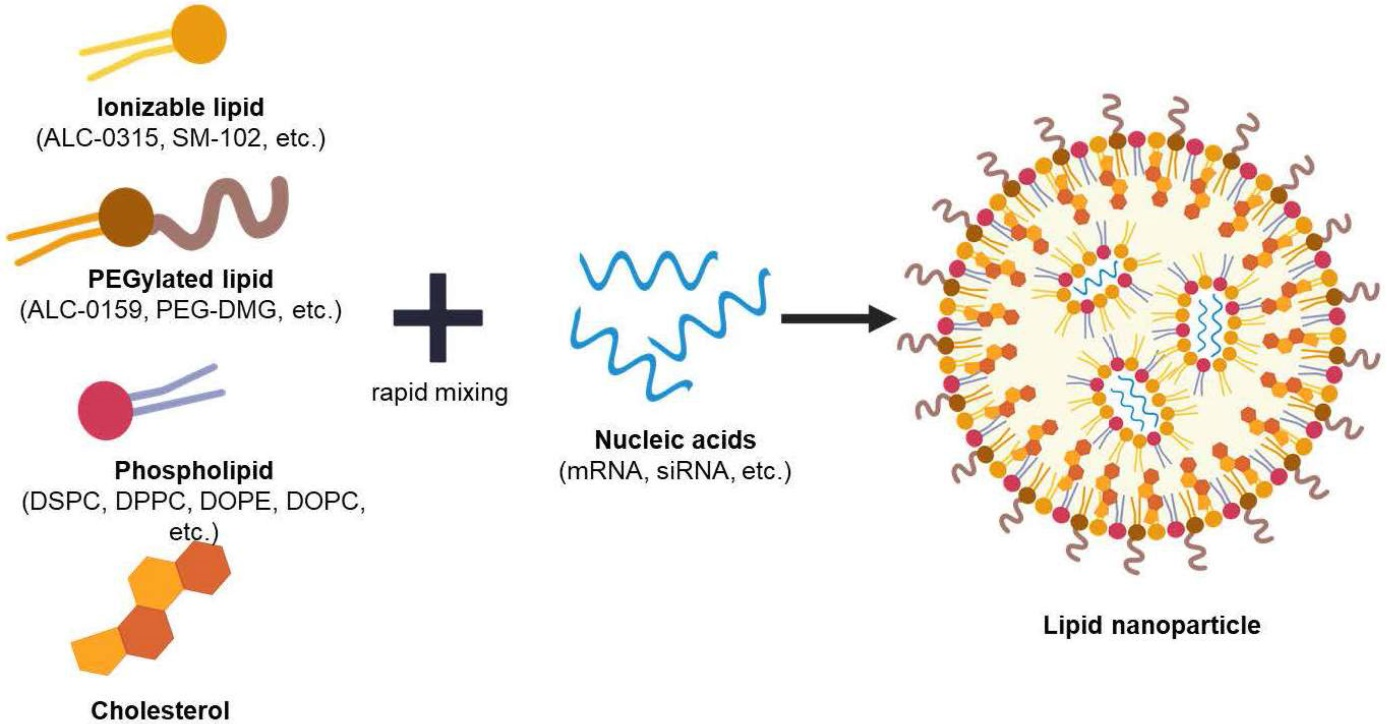
Illustration of lipid nanoparticle and its compositions. Created with BioRender.com.

The idea of using mRNA as a novel therapeutic drug first took place in 1989 by Vical Incorporated a start-up company in San Diego. The company first published its research demonstrating mRNA encapsulation into liposomes and succeeded in transfecting mRNA into various eukaryotic cells 15. Afterwards, various studies have been done to properly deliver mRNA across the cell membrane into cytosol, because mRNA directs the synthesis of proteins in the cytoplasm using various nanoparticles such as polymeric particles and liposomes 2, 16, 17. Recently, the use of LNPs as mRNA delivery vehicles has become a leading technology in the application of vaccines 18-20. Loading mRNAs into LNPs is stable, leading to a successful delivery into cells 21. The LNPs typically consist of four different components with a unique role shown in both **Figure 2** and **Table 1**.

### Ionizable lipids

The ionizable lipids determine the potency of the LNP because it has a crucial role in encapsulating mRNA or other different types of nucleic acids 22. In general, cationic lipids contain alkylated quaternary ammonium groups for which the charge of the lipids remains the same in different pH. On the other hand, ionizable lipids form a positive charge at a low pH (<6.0) because the free amine is protonated as the pH decreases. When the pH changes back to physiological condition (pH ~7.4), the charge of the ionizable lipids becomes less positive or neutral 23, 24. This characteristic of ionizable lipids offers several major advantages in the delivery of mRNA. First, the switching of the lipid charge at a different pH enables a high encapsulation efficiency of the mRNA because the positive charge of the lipid interacts with the anionic mRNA at a low pH 25-28. Therefore, formulating LNPs using ionizable lipids offers less positive or neutral charge at a physiological pH, which not only enhances the biocompatibility of the nanoparticles but also helps prevent the nonspecific binding of anionic biomolecules 26. The capability of the change in charge of the ionizable lipids depending on the environmental pH is recognized as a key component of the endosomal escape of LNPs. As endosomal maturation begins, the low pH inside the endosome turns ionizable lipids to a positive charge. These positively charged ionizable lipids interact with the negatively charged inner endosome membrane forming a non-bilayer structure of a hexagonal (H_II_) phase. As membrane fusion occurs, the entrapped nucleic acids in the LNPs escape and are released into the cytosol 29. However, it has been reported that less than 2-3% of nucleic acids escape from the endosome and are released into the cytosol 30, 31. Therefore, improving the percentage of RNA release into the cytosol is one of the keys to open the full potential of LNPs.

### Phospholipids and cholesterol

The other components of LNPs are the “helper lipids,” which are phospholipids (i.e., DSPC, DOPE, etc.) and cholesterol. In general, these helper lipids not only provide structural stability of the nanoparticle, which improve the biodistribution of LNPs and enhance the delivery efficacy by promoting intracellular uptake and cytosolic entry 32, 33. Additionally, depending on the types of phospholipids, it can lead to disruption of the lipid bilayer, which promotes endosomal escape 34-38.

One of the commonly used phospholipids is a phosphatidylcholine (PC), such as 1,2-distearyol-sn-glycero-3-phosphocholine (DSPC) and hydrogenated soybean PC (HSPC). DSPC is clinically applied such as siRNA therapeutics (Patisiran) and mRNA vaccines against SARS-CoV-2 (mRNA-1273 and BNT126b2) 5, 33. DSPC contains a saturated acyl chains, containing one or more double bonds, in the tail of the lipid and a relatively larger head group, which forms a cylinder-shaped geometry. Furthermore, the high melting temperatures (T_m_) value of this lipid provides highly stable LNP structure 39-41. Due to its high stability, it inhibits membrane fusion with the endosomal membrane, which inhibits endosomal escape. On the other hand, DOPE is considered as a fusogenic lipids. It contains two unsaturated acyl chains, containing hydrocarbon chain with single bond only, in the tail and relatively smaller head group, which forms cone-shape geometry 39, 42. These unsaturated acyl chain lipids have low melting temperature (T_m_) values and stabilize the non-bilayer hexagonal II (H_II_) phase. In the physiological temperature, DOPE forms a non-lamellar lipid phase due to the inverted hexagonal (H_II_) phase. This enables membrane fusion, bilayer disruption, that leads to endosomal escape 36, 43, 44. Additionally, DOPE is reported to enhance the transfection efficacy when present in cationic lipid formulations by facilitating membrane fusion 44-46.

Cholesterol is also used in LNP development as it enhances particle stability by regulating membrane integrity and rigidity 33. According to Patel et al., cholesterol has an important role in enhancing gene transfection and biodistribution of mRNA-LNPs due to the analog with C-24 alkyl phytosterols. Therefore, the study by Patel et al. reports that maintaining high transfection requires 1) the polarity of the hydroxyl group, 2) the sterol ring flexibility, and 3) length of alkyl tail 47. Kotoucek et al. also provide an insight into the role of cholesterol in the formulation of liposomes by formulating liposomes with various molar ratios of cholesterol. Because cholesterol was mixed with DSPC with an increasing molar ratio (10 ~ 50 mol %), the liposome size decreased, indicating the amount of cholesterol affects the size of the liposomes 48.

### PEGylated lipids

The PEGylated lipids have various roles in LNP formulations and mRNA delivery 49-52. The structure of the PEGylated lipids contains a hydrophilic head and hydrophobic tail. The PEGylated lipids have a wide molar mass range from 400 Da to 50 kDa. Therefore, it is important to use an appropriate molar mass according to the application. The amount of PEGylated lipids in LNP formulations determines the size and zeta potential, which are also important properties that affect the delivery efficacy 50. The large molar mass of PEG, such as 20 to 50 kDa, is applied in the usage of drugs with low molar mass (e.g. oligonucleotides, siRNA, small molecules, etc.). The use of a large molar mass of PEG increases the size of the drug carrier, enabling it to avoid renal clearance. On the other hand, a lower molar mass of PEG, such as 1 to 5 kDa, is used in larger drugs: antibodies and nanoparticle drugs 49.

Furthermore, there are many research reports that PEGylated lipids enhance stability and decrease the aggregation of LNPs 27, 33, 49. Thus, PEGylated lipids enhance the blood circulation time and protect the LNPs surface, decreasing the kidney's clearance. PEGylated lipids also protect LNPs from uptake by the mononuclear phagocyte system (MPS) 51-53. As PEGylated lipids prevent aggregation and control the size of LNPs, they overall increase the stability with a potential decrease of ApoE adsorption and particle fusogenicity 54-57. The “stealth effect” through PEGylated lipids decreases protein adsorption and the cellular uptake and transfection capacity of LNPs 58. To overcome this so-called “PEG-dilemma,” the length of PEGylated lipids needs to be considered as the shorter acyl chains (C8-14) quickly spread out of the LNP than the longer acyl chains (C16-C24), which achieve a higher transfection efficiency 59-63.

Though PEGylated lipids are one of the crucial and useful components of LNPs, PEG immunogenicity and anti-PEG antibodies need to be considered in developing LNP-based vaccines and therapeutics. According to recent studies, both SARS-CoV-2 mRNA LNP-based vaccines, mRNA-1273 (Moderna) and BNT126b2 (Pfizer/BioNTech), induced the amount of anti-PEG IgM and anti-PEG IgG in human samples 64, 65. Both studies show a common detection of anti-PEG antibodies before the vaccination due to previous exposure to PEG from cosmetics and PEG-containing medicines. However, both mRNA-1273 and BNT126b2 vaccines significantly induced anti-PEG IgM and anti-PEG IgG after the administration. Furthermore, studies showed that mRNA-1273 induced more anti-PEG antibodies than BNT126b2. This result could have been affected by several reasons. 1) The type of PEG's terminal group and 2) the shedding rate of PEGylated lipids from LNPs 65-67. There are different terminal groups of PEG, such as methoxy, hydroxy, etc. Clinically, most PEGylated drugs use methoxy terminal group PEG (mPEG). Compared to hydroxy-PEG (HO-PEG), mPEG induces higher PEG immunogenicity 64-66, 68. Also, most PEG-specific antibodies identify the repeated ethylene oxide subunits, the backbone of the molecules. Although both mRNA-1273 and BNT162b2 apply different PEG structures, they both contain methoxy terminal group PEG. Therefore, it is unlikely the structure of the PEG that affects the inducing amount of anti-PEG antibodies but rather the higher dose of mRNA-1273 (100 μg) administered compared to BNT126b2 (30 μg). Another factor that causes PEG immunogenicity is the shedding rate of PEG 67-69. The anti-PEG antibodies are induced depending on the shedding rate of the PEGylated lipid from LNPs. The shedding rate is dependent on the length of the acyl chain. A short acyl chain leads to fast shedding of LNP, which induces less PEG immunogenic, whereas a longer acyl chain leads to slower shedding, which induces a more immunogenic response 67, 69. Several studies mentioned regard to PEG immunogenicity and anti-PEG antibodies. There is still a need for clinical relevance in PEG-specific antibodies induced by mRNA LNP-based medications and vaccines, as well as exploring an alternative to PEG in developing mRNA LNP-based vaccines and therapeutics.

### Various production methods for LNPs

### Thin-film hydration

Cationic liposomes were the first delivery carrier used in mRNA vaccines 18. Thin-film hydration or the Bangham method is one of the most commonly used techniques for liposome production 70. This passive encapsulation approach is produced as the phospholipids spontaneously self-assemble into vesicles 71, 72. Because the lipids are dissolved in an organic solvent, such as ethanol or chloroform, the lipids are then evaporated through a rotary evaporator, leading to a thin lipid layer formation. As the thin layer is hydrated using an aqueous buffer solution loaded with nucleic acids, the hydrophobic and hydrophilic parts of the lipids are self-assembled. Thus, it leads to the formulation of large multilamellar vesicles (MLVs) that are very heterogeneous with a size of micrometers 73, 74. The advantages of using thin-film hydration methods are 1) it is the simplest production procedure to prepare, 2) it does not involve in using expensive, complex lab equipment, and 3) high pressure or temperature is not needed in maintenance.

However, the size of the vesicles must be reduced and homogenized through extrusion or sonication of the MLVs to form small or large unilamellar vesicles (SUVs and LUVs) for effective therapeutic administration 75-77. The extrusion process to reduce the size of the vesicles is done by repeatedly forcing the heterogeneous suspension through a filter of the desired size. Sonicators (probe or bath) is another method used to reduce the vesicle size. However, sonication for size reduction provides less control over the size result than extrusion 78, 79. Though this method is commonly used in vesicle production, the complex steps of the manufacturing method and its labor-intensiveness make this production lack scalability and result in high costs 80-82. Another limitation of using the thin-film hydration method is the low encapsulation efficiency in general. An excess amount of lipids is used in the production process to improve the encapsulation efficiency, which leads to an increase in the cost and toxicity 79. As the batch size increases, the volume of the organic solvent will also increase, which would take several hours to evaporate the organic solvent, making it time-consuming at a large scale 71. In addition, a mass amount of production would be difficult using extruders, which may lead to filter tears and clogging of the membrane that will result in a large batch failure and product loss 80.

### Ethanol injection

Ethanol injection is another general technique that forms unilamellar liposomes. In this method, lipids are dissolved in ethanol and injected at a fast rate into an aqueous buffer under stirring, containing drug or the compound for encapsulation, where the volume is relatively higher than the lipid solution, rapidly forms vesicles 83, 84. One of the advantages of using ethanol injection methods is the simple and convenient procedure. Gouda et al. reports the production of homogenous SUVs can be formulated when the ethanol percentage is less than 7.5% of the total volume (v/v) through ethanol injection method 84. This production method is one of the earliest alternatives to the thin-film hydration method, in which a sonication procedure is not needed 83. This technique was first introduced and developed in 1973 by Batzri and Korn 85. The self-arranged vesicles are formed as the injected lipid solutions are quickly diluted with an aqueous buffer due to the increase of polarity in the mixed solvent. The size of these vesicles is controlled through the lipid concentration, stirring rate, injection rate, and the types of lipids used 85.

The other type of ethanol injection method called “crossflow injection” was developed as another way for the use of the mass production of liposomes 84. This alternative device consists of two stainless steel tubes in a cross-shaped form with a small injecting hole at the intersection between the tubes. Through the crossflow tube, the ethanolic solution containing lipids is injected into a buffer solution by changing the pressure of the nitrogen regulator device forming liposomes. These various ethanol injection methods use high lipid concentrations, and these manufacturing production methods are not generally used due to the large size of heterogeneous nanoparticle production and low mRNA encapsulation efficiency 86. Additionally, reproducibility on a large scale is challenging to accomplish because the use of the sonication procedure can degrade lipids and drug contents. The degradation of lipids and drug contents were reported as the samples are overheated through the sonication procedure 85, 87.

Nevertheless, these conventional liposomal methods mentioned are still widely used due to their simple implementation 88. However, the lack of reproducibility, scalability and encapsulation efficiency holds them back from progressing further into clinical translation efficiency 89. New production methods have been reported to overcome these challenges, which will be discussed below.

### T-junction mixing

A lipid-based drug produced by T-junction mixing was introduced by Hirota et al. in 1999 90. Compared to the macroscopic mixing methods such as vortexing and pipetting, T-junction mixing produces reproducible and controllable nucleic acid-loaded lipoplexes 91. Therefore, this method is the general choice for large batches of mRNA-LNPs for commercial production. T-junction produces rapid mixing through the injection of two different solvents as each inlet collides, which causes a turbulent flow. Moreover, the constant speed of the mixture is important. Though there are limited data on the different factors that could influence the formulation of LNPs, Kulkarni et al. demonstrated the flow rate ratio of the mixing solution influences the size and PDI value when formulating LNPs 92. In that study, T-junction mixing was used to produce LNPs encapsulating inorganic hydrophobic nanoparticles, iron oxides. The results show that the size of the LNP decreased with the increase in the total flow rate. When the total flow rate was 10 mL min^-1^, the size of the LNP was observed to be 75 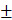 6 nm, while the size of the LNP was 36 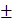 2 nm when the total flow rate was 40 mL min^-1^. The value of PDI was also affected by the flow rate because the PDI value was higher at a lower flow rate compared to the low value of the PDI at a higher flow rate 93. This result indicates that the control of the flow using T-junction mixing can impact the characteristics of the nanoparticle formulation.

Though there are few productions of mRNA-LNPs using T-junction mixing at the laboratory scale, there are some studies are being done regards to the encapsulation efficiency as well as the particle formulation size and morphology. Goswami et al. used mannosylated lipid nanoparticles to potentially enhance the uptake by antigen-presenting cells (APCs) and encapsulated mRNA 94. The LNPs were composed of DSPC, cholesterol DLin-DMA and DMG-PEG2000 (10:48:40.2 mol %) and prepared in ethanol. The ethanol dilution method using T-junction was used. Then the lipid content in ethanol was mixed with mRNA in a 100 mM citrate buffer (pH 6) with a nitrogen to phosphate (N/P) ratio of 8. The resulting size of the particles was 129.9 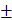 0.98 nm with a 0.1 PDI value. The resulting encapsulation efficiency was 88.40 %, successfully encapsulating 11.78 μg mL^-1^ of the mRNA. Additionally, Chen et al. demonstrated a similar T-junction mixing setup to formulate LNPs with various encapsulated nucleic acids, such as CpG, siRNA, mRNA, and pDNA 95. For the mRNA encapsulation, DLin-MC3-DMA, DSPC, cholesterol, and PEG-DMG (50:10:38.5:1.5 mol %) were dissolved in ethanol and mixed with mRNA dissolved in 25 mM acetate buffer at pH 4 through T-junction mixing. The ethanolic and aqueous solutions were mixed at a 1:3 (v/v) ratio with a final flow rate of > 10 mL min^-1^. The exact size and the encapsulation efficiency of the nanoparticles were mentioned. However, it was indicated that the range of the nanoparticle sizes was 46 ~ 50 nm with a PDI value less than 0.1. It was also mentioned that LNPs exhibiting < 86 % nucleic acid entrapment were not used for the further experiments 95. Lazzaro et al. also applied T-junction mixing in their fabrication of mRNA-LNPs for CD8 T-cell priming upon self-amplifying mRNA vaccination. In this experiment, mRNA was encapsulated with DLin-DMA, DSPC, PEG-DMG2000, and cholesterol (40:10:2:48 mol %) with an N/P ratio of 8 using T-junction mixing. The resulting nanoparticle sizes were < 200 nm with a PDI value < 0.15. The encapsulation efficiency was > 85% 96, 97.

These previous works show that mRNA-LNPs can be formulated using T-junction mixing. This method generally offers a higher encapsulation efficiency than encapsulation through conventional liposomal methods. However, the high flow rate requirement for rapid mixing through T-junction is not preferred on a laboratory scale but for large-scale production. Moreover, the mixture is dependent on the channel length and the contact surface area. Therefore, the decreased influence of mass transport can be caused by a short mixing time by T-junction mixing, which leads to lipid aggregation and heterogeneity of the nanoparticle formulation 98. Though there are several reports on LNP production through T-junction mixing, further studies are needed on identifying the various factors that affect the LNP formulation.

### Microfluidic mixing

Typical operating microchannel conditions have a laminar flow with slow molecular diffusion through the channel. The absence of a turbulent flow makes it difficult to mix solutions effectively. That is why the addition of the staggered-herringbone structure to the fluidic channel was implemented and introduced by Stroock et al. in 2002 99. Compared to the simple and smooth channel of a typical microchannel, the additional structure to the fluidic channel promotes the reduction of hydrodynamic dispersion, enabling an effective mixing of solutions. This chaotic mixing method of microfluidics was pioneered in developing LNPs by the Pieter Cullis research group and later commercialized by Precision Nanosystem 23, 100, 101. Additionally, it has been reported that staggered-herringbone structured microfluidic chips can be simply extensible to produce large batches 102. The development of pattern structured microfluidic chips has improved the control of the mixing procedure leading to the control of homogenous particle size and distribution and a higher encapsulation compared to the bulk methods 103. The rapid mixing offered through the staggered-herringbone structure suddenly increases the polarity of a mixture, which causes supersaturation that results in LNP formation 103, 104. It has been reported that controlling the total flow rate (TFR) and the flow rate ratio (FRR) can affect the size and the size distribution of the LNPs, for which the structured microfluidic chip is a suitable application in LNP production 100, 103, 105. Recently, Moderna has reported the size regulation of mRNA-LNPs through TFR control 106, 107. The mRNA-LNP was formulated using the novel synthesized ionizable lipid H. Lipid H, DSPC, cholesterol, and PEG2k-DMG with molar ratios of 50:10:38.5:1.5 were dissolved in ethanol mixed with mRNA buffer solution through a microfluidic system at different TFRs. The TFRs ranged from 0.5 mL min^-1^ to 12 mL min^-1^, and it was observed that the particle size decreased as the TFR was increased. Furthermore, Shepherd et al. have also demonstrated the scalable production of mRNA and siRNA LNPs using a parallelized microfluidic device (PMD) 102. The PMD contains an array of 128 mixing channels that simultaneously operate. It can produce over a 100-fold production rate compared to a single-channel microfluidic chip. The mRNA-LNPs produced by PMD contain C12-200, DOPE, cholesterol, and PEGylated lipid with luciferase mRNA at a flow rate of 18.4 L h^-1^. The results showed the ability to reproduce the size of the mRNA-LNPs that were > 140 nm with a low polydispersity.

Though there are many advantages of both T-junction and microfluidic mixing, one of the drawbacks is the requirement of a large amount of organic solvent in manufacturing production as shown in **Table 2**. Additionally, using ethanol to dissolve lipids can bring about limitations in the types of lipids chosen 23. Regardless, LNP production through microfluidic mixing methods became a major platform encapsulating various types of nucleic acids such as siRNA and mRNA. There are various microfluidic chip designs and different particle formulations, making it difficult to compare directly. However, the rapid mixing production methods such as T-junction and microfluidic devices have accomplished efficient and reproducible mRNA-LNPs. Scalability can be easily accomplished from a small laboratory to a large manufacturing scale 102. Therefore, the production of mRNA-LNPs through microfluidic mixing has shown potential for opening a new pathway for nucleic acid-based therapeutics.

## Recent advances in LNP-based RNA therapeutics in clinics or clinical trials

### Clinical stage LNP-based RNA therapeutics

As various gene therapies enter clinical trials, LNPs have been considered as the most optimized and applicable delivery system for nucleic acids such as siRNA and mRNA because negatively charged nucleic acids interfere with the delivery to cell membranes and are degraded by endogenous nucleases in the body, making efficient delivery difficult without the aid of LNPs 88, 108. Patisiran (ONPATTRO®), the first Food and Drug Administration (FDA) approved LNP-formulated siRNA drug, has reduced the formation of transthyretin protein in the liver and has been used to treat hereditary transthyretin-mediated amyloidosis since 2018. Amine head groups of the ionized lipid by 4-(diethylamino)-butanoic acid (DLin-MC3-DMA) optimized for siRNA delivery has had an important role in the drug 70. Givosiran (GIVLAARI^TM^), which received FDA approval in 2019, is a siRNA that targets the ALAS1 gene in hepatocytes and is prescribed for patients with the hereditary disease acute hepatic porphyria 109, 110. The most recent topic is the Severe Acute Respiratory Syndrome Coronavirus 2 (SARS-CoV-2) infection, declared a pandemic by the World Health Organization (WHO). Representatively, Pfizer and BioNTech's BNT162b2 and Moderna's mRNA-1273 are LNP-formulated mRNA vaccines that have received emergency use authorization (EUA) by the FDA in December 2020. Most recently, on August 23, 2021, Pfizer/BioNTech's BNT162b2 was formally approved by the FDA for people 16 years of age or older or by the European Medicines Agency (EMA) for people 12 years of age or older with the official name “Comirnaty” (mRNA vaccine for COVID-19). Additionally, on January 31, 2022, Moderna's mRNA-1273 was the second approval of a mRNA vaccine for COVID-19 by the FDA for people 18 years of age and older or by the EMA for people 6 years of age and older with the official name “Spikevax”.

### mRNA vaccine platforms

Unlike DNA-based vaccines, mRNA vaccines have less potential risk of insertional mutagenesis because mRNA vaccines are non-infectious, non-integrating platforms when compared to conventional vaccine platforms containing subunits, attenuated, inactivated, and killed pathogens. *In vitro* transcribed mRNA (IVT mRNA) is emerging as a potential replacement for conventional vaccines due to its advantages of faster, cheaper, and scalable manufacturability due to its more stable, translatable, and high yield productivity after transcriptional reactions 19. The developed IVT mRNA is composed of four structurally modified forms to optimize its high translation efficiency and stability: 1) 5'-cap, 2) poly(A) tail, 3) 5' or 3'-untranslated regions (UTRs), 4) Open reading frame (ORF) 5, 111. First, the 5'-cap modification mediates the binding of the decapping enzyme (DCPs) by attaching eukaryotic translation initiation factor 4E (EIF4E) to the cap analogues. Second, the poly(A) tail must be adjusted in length to affect the translational stability, and deadenylation is inhibited by recombinant poly(A) polymerase. Third, the 5'-UTR including internal ribosomal entry sites (IRES) improves the protein translation efficiency, and the 3'-UTR with an alpha or beta-globin sequence improves the IVT mRNA stability. Fourth, codon optimization in protein translation is known to be important for efficiency and acceleration. However, IVT mRNA has a limitation in direct clinical use due to the difficulty of cell membrane penetration from its anionic property 112. Various clinical trials for mRNA-based vaccines are actively underway in infectious diseases 113, and other rare diseases.

**Figure 4:**
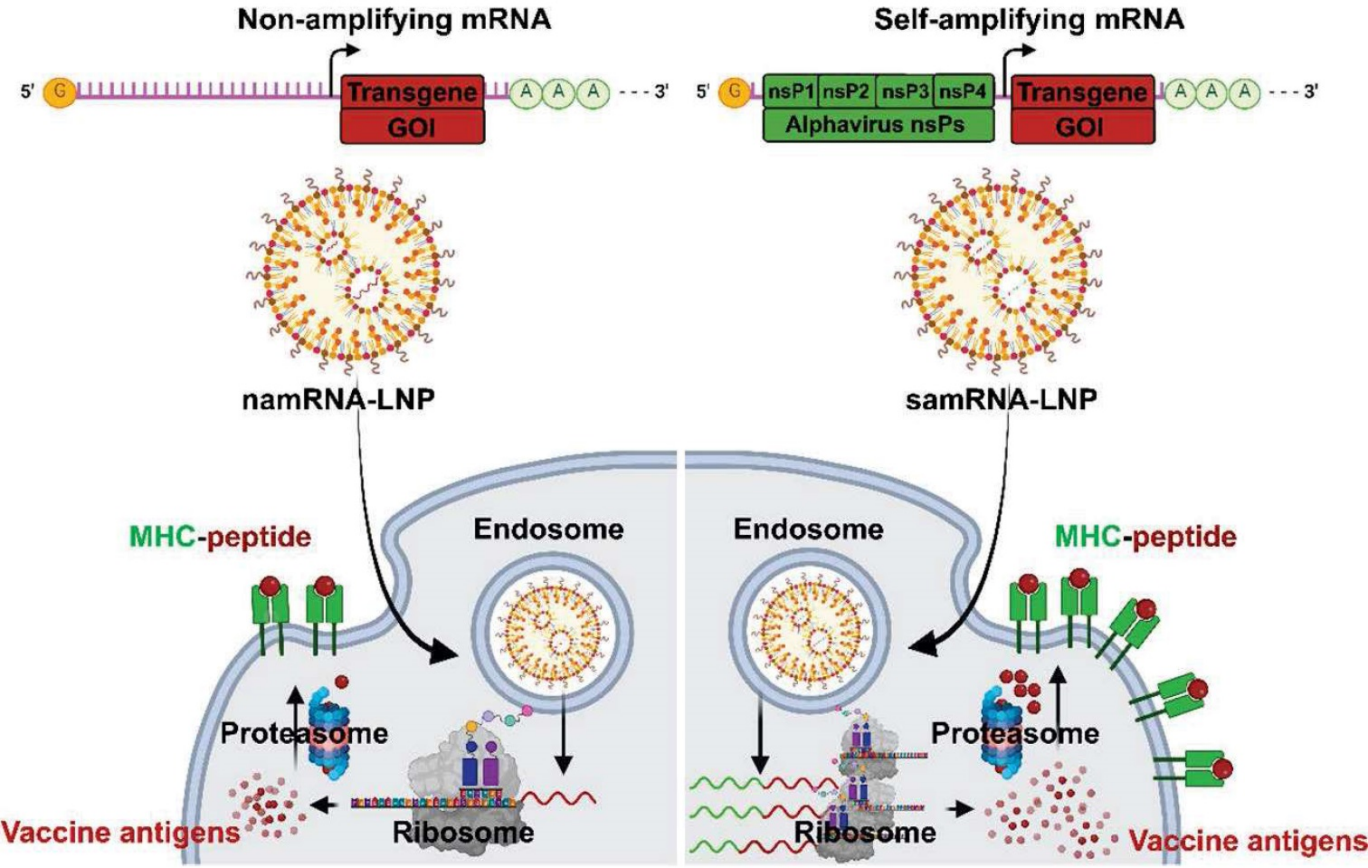
Schematic mechanism of vaccine antigens with non-amplifying mRNA (namRNA) versus self-amplifying mRNA (samRNA). GOI: gene of interest; LNP: lipid nanoparticle; MHC: major histocompatibility complex; nsPn: non-structural polyprotein number.

Over the past decade, LNP formulations have been used to deliver various mRNA vaccine platforms. Typically, three types of RNA vaccine platforms have been used: self-amplifying messenger RNA (samRNA), non-amplifying messenger RNA (namRNA) vaccines 114 and circular RNA (circRNA) as shown in **Figure 4**.

The samRNA vaccine is based on the alpha virus genome and replaces the gene encoding “the antigen of interest” with the “the antigens of the vaccine” to generate a large amount of “the antigen of the vaccine” through intracellular replication by RNA replicon even with a very small amount of the mRNA vaccine 115. Unlike samRNA, namRNA is a typical mRNA that is generally considered non-amplifying and does not have an RNA replicon. The namRNA was also used as a vaccine for infectious diseases or cancer and has economic advantages but has partial disadvantages in its low stability and ability to generate antigens, which are mRNA transcripts 19, 116. Because samRNA is larger than namRNA and has an anionic charge, a cationic or ionizable LNP with a size of ~100 nm is used to facilitate cellular uptake and cytoplasmic release 26. Clinical trials of samRNA vaccines are being actively conducted for various diseases. For the COVID-19 vaccine, the samRNA vaccine was first completed in a phase 1 / 2 clinical trial by the Imperial College London research team from 2020 to January 2021 (ISRCTN17072692). The benefits of samRNA were demonstrated using doses ranging from 0.1 to 10 μg, much smaller than the Pfizer/BioNTech (BNT162b2, 30 μg) and Moderna (mRNA-1273, 100 μg) vaccines. In addition, ARCT-021 from the Duke-NUS School of Medicine and Arcturus Therapeutics Inc., in a phase 1 / 2 clinical trial (NCT44800957), was found that multiple dose levels from 1 to 10 μg in healthy people aged 18 to 80 years, excluding pregnant and breastfeeding women, were evaluated as safe, tolerable, and immunogenic 116.

In addition, an RNA vaccine platform that is attracting attention is the single-stranded, loop-type circRNA. It is stable from nuclease-mediated degradation due to the lack of a 5'-cap or 3'-poly(A) tail, and has no stop codon, enabling continuous and high-efficiency protein translation 117, 118. These circRNAs are already being used as targets for therapeutic approaches in various cancers, cardiovascular diseases, and central nervous system diseases 119.

### mRNA-LNP vaccines for infectious diseases

As of September 07, 2022, multiple clinical trials for mRNA-LNP vaccines for various infectious diseases are ongoing with a total of 216 active (recruiting or not), suspended, terminated, withdrawn, or completed trials (https://clinicaltrials.gov/ct2/results?cond=Infectious+disease&term=mRNA+vaccine&cntry=&state=&city=&dist=). We summarized clinical trials with mRNA-LNP against infectious diseases as shown in **Table 3**.

### Rabies

CureVAC AG RNAactive® CV7201 is a lyophilized, namRNA candidate vaccine encoding the rabies virus glycoprotein (RABV-G) and the ionizable cationic protein protamine. From October 2013 to January 2016, a dose of 80 to 640 μg of CV7201 was administered intradermally or intramuscularly by a needle syringe or needleless syringe device in 101 healthy people aged 18-40 years (NCT02241135). Seven days after vaccination, most showed local reactions and 78% systemic reactions. Needle-free delivery showed varying levels of neutralizing antibody induction, but the needle-syringe delivery did not 120. Since October 2018, CureVAC AG's new mRNA-LNP, CV7202, has been undergoing a phase 1 clinical trial in 53 participants aged 18-40 years (NCT03713086). Two doses of 1 or 2 μg of CV7202 induced a higher rate of rabies-neutralizing antibody response than a single dose of 5 μg with a high reactogenicity in all recipients 121.

### Influenza

Moderna's mRNA-1440 (VAL-506440) and mRNA-1851 (VAL-339851) are vaccines encoding from Haemagglutinin H10N8 and H7N9, respectively 122. The inoculation of the mRNA-1440 vaccine administered as two doses of the vaccine 3 weeks apart showed 78.3% inhibition of haemagglutinin at 100 μg in healthy people aged 18-64 years (NCT03076385) and 96.3% inhibition by the inoculation of mRNA-1851 at 50 μg in healthy people aged 18-49 years (NCT03345043) 123.

### Zika virus

Moderna's mRNA-1893 against Zika virus is an mRNA-LNP vaccine encoding the prM-E (pre-membrane and envelope) protein 124. In a Phase 1 clinical trial conducted from 2019 to April 2020, a 100 μg dose level of mRNA-1893 induces a potent neutralizing Zika virus antibody response in both patients with and without flavivirus infection in 120 healthy people aged 18-49 years (NCT04064905). However, two dose levels (one-dose or two-dose schedules) of mRNA-1893 are being evaluated compared to a placebo in 800 healthy adults in a phase 2 trial based on the results initially observed at the 30 μg level in the phase 1 trial 125, 126.

### Cytomegalovirus

Two mRNA vaccine candidates (mRNA-1647, mRNA-1443) against Cytomegalovirus (CMV) completed a phase 1 clinical trial from November 2017 to October 2020, in 181 healthy participants aged 18-49 years (NCT03382405). mRNA-1647 consists of six mRNAs of two antigens, five encode pentameric complexes and one encodes the full-length membrane-attached glycoprotein B, and mRNA-1443 encodes pp65 from T cells of the CMV antibody 127. High neutralizing antibody responses (geometric mean titers, GMTs) in CMV seronegative and positive groups were achieved with excellent tolerability and no serious adverse effects 1 month after the third vaccination. Based on these results, in a phase 2 clinical trial, 452 healthy participants aged 18-40 years were vaccinated with 3 doses of 50, 100 and 150 μg once a month, respectively, in January 2020. Both the CMV seronegative and positive groups showed a dose-dependent increase in the neutralizing antibody GMT for 7 months after the first administration 128. Based on this, mRNA-1647 (100 μg dose) entered a phase 3 clinical trial in 2021 114.

### SARS-CoV-2, COVID-19

**Figure 5:**
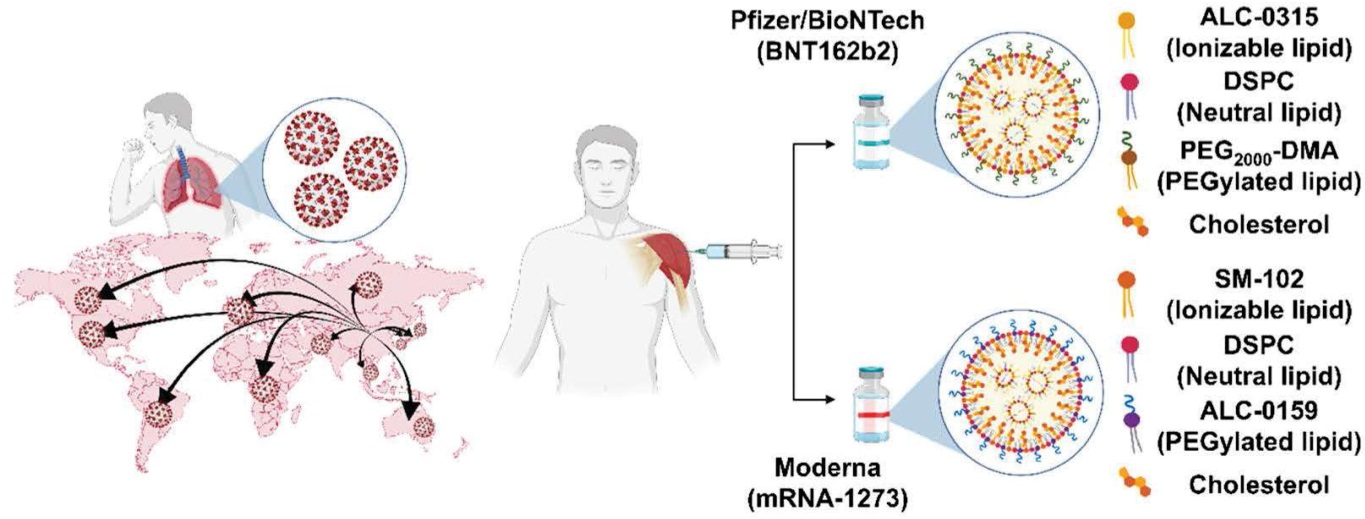
Development and representative lipid compositions of the most advanced mRNA vaccines with COVID-19: Pfizer/BioNTech (BNT162b2) and Moderna (mRNA-1273).

In December 2019, COVID-19 was identified. There were no vaccines for COVID-19 infection, so the incidence and mortality rates began to accumulate rapidly 129, 130. Therefore, as the clinical trials of new vaccines received urgent approval, various companies started developing the vaccine. Among them, Pfizer/BioNTech and Moderna reported that their mRNA vaccines against COVID-19 have shown a strong effect on disease prevention 131 seen in **Figure 5**. In the vaccines from both companies, the mRNAs encode the spike (S) protein of SARS-CoV-2 and translate into S proteins that serve as antigens and contribute to the development of the immune response of COVID-19.

In October 2020, Pfizer and BioNTech first validated the results of a comparative clinical trial of BNT162b1, encoding the receptor-binding domain (RBD) of the S protein in SARS-CoV-2, and BNT162b2, encodes a full-length S in an LNP formulated and nucleoside-modified RNA (modRNA). BNT162b2 showed a lower systemic reactivity and toxicity in 43,998 participants aged 18-85 years in the phase 1 clinical trial 132. Two months later, on December 11, 2020, it was approved for EUA by the FDA, demonstrating a 95% efficacy and safety in participants aged 16-85 years through phase 2 and 3 clinical trials 133, including adolescents aged 12-15 years 134. In pregnant women, there were no problems with safety, and no effect on infant weight or survival 135, 136.

Moderna's mRNA-1273 is a nucleoside-modRNA-based vaccine in an LNP formulation encoding the stabilizing S protein by replacing two prolines (S-2P, at 86 and 987). The structural rearrangement of the fusion S2 subunit is prevented in heptad repeat 1 located at the top of the S-2P. From March 2020, mRNA-1273 showed strongly biased expression of Th1 cytokines in people aged 18-71 years through a phase 1 clinical trial and a rapid S-2P antibody binding reaction (NCT04283461) 137, 138. The FDA approved EUA for the COVID-19 pandemic in December 2020, validating the safety and efficacy of 94.1% at preventing COVID-19 through a 2-part phase 3 clinical trial (NCT04470427) 139. In addition, mRNA-1273 showed antibody activity for all ages for 180 days after the second dose (209 days) through the RBD ELISA and Neutralization assay 140.

After the outbreak of the Omicron (B.1.1.529) mutation in November 2021, the fourth vaccination became inevitable despite receiving the second and booster vaccinations. In an open label, non-randomized clinical study, mRNA-1273 showed 9.73-fold the IgG titer and 7.23-fold the neutralizing titer in the participants receiving fourth dose vaccination compared to the participants who 5 months after the booster vaccination aged 18 years and older (NCT NCT05230953). Also, BNT162b2 showed 7.01-fold the IgG titer and 10.73-fold the neutralizing titer in the participants receiving fourth dose vaccination (NCT05231005) 141.

LNPs in vaccines against mRNA COVID-19 are similar to four major components, although the molar lipid composition differs 5, 113. Because the mRNA-LNPs of both companies have the anionic property of mRNA, ionizable lipids (Pfizer (ALC-0315); Moderna (SM-102)), which are positively charged (protonation) at low pH, are used to construct the LNPs. The phospholipid, 1-2-distearoy-sn-glycero-3-phosphocholine (DSPC), and cholesterol are helper lipids that can improve the stability, tolerability, and biodistribution. PEGylated lipids (Pfizer, ALC-0159 (PEG-DMA); Moderna (1,2-dimyristoyl-rac-glycero-3 methoxypolyethylene glycol-2000) (PEG-DMG)) increase the enhanced permeability and retention (EPR) effect to ensure stability and prevent aggregation during storage 20, 131 as seen in** Table 4**.

In the mRNA part, to prevent degradation and stabilize the mRNA structure, 1-methyl-pseudouridine (m1Ѱ), one of the naturally occurring converted uridines, in modRNA was combined to induce secondary structure changes in the mRNA to correlate with high protein translation. Another third mRNA candidate vaccine 19, 131, CvnCov from CureVAC AG, did not meet the FDA approval criteria with an overall efficacy of 48% in the 2b / 3 clinical trial (NCT04652102) between December 11, 2020 and April 12, 2021 142-144. It has been suggested that one of the reasons was that, unlike the Pfizer-BioNTech or Moderna vaccine, it does not contain m1Ѱ.

Local events such as pain at the injection site, edema, erythema, or systemic events such as fever, headache, vomit, and chills are commonly reported after vaccination against COVID-19. Specifically, on May 17, 2022 for BNT162b2 or on March 29, 2022 for mRNA-1273, the FDA revised the Patient and Provider Fact Sheet because severe systemic outbreaks of anaphylaxis, thrombosis, and myocarditis were reported as COVID-19 vaccination expanded worldwide. As of March 14, 2022, the Vaccine Adverse Event Report System (www.vaers.hhs.gov, VAERS) has received 4,626 (2,757 for BNT162b2, 1,869 for mRNA-1273) reports of myocarditis or pericarditis among people 3 years of age or older who have received the both COVID-19 vaccine. In addition, 11,997 (9,211 for BNT162b2, 2,786 for mRNA-1273) cases reported by European Union/European Economic Area (EU/EEA) EudraVigilance system (https://www.ema.europa.eu/) 145. Most have been reported after vaccination with the mRNA COVID-19 vaccine (Pfizer-BioNTech or Moderna), especially in male adolescents and young adults, but there is an increasing trend in females as well 146-149. Although the correlation between mRNA-LNP-based vaccines against COVID-19 and myocarditis has not yet been clearly elucidated, the strongest hypothesis is that cationic lipids in the LNP component induce antibody-mediated cytokine expression and activate aberrant apoptosis 106, 150. Moreover, among the lipid components, PEGylated lipids can activate the host immune response and stimulate the complement system to induce hypersensitivity reactions after systemic or local administration. Due to these stability problems, studies that can replace PEG are needed and are in progress 20.

Recently, rapid and useful vaccines have been developed against COVID-19, but many mutant variants are emerging 151. To date, the five representative variants known are Alpha (B.1.1.7), Beta (B.1.351), Gamma (P.1), Delta (B.1.617.2) and Omicron (B.1.1.529) which are the forms of the receptor binding domain (RBD) in the S protein 152-156. If its protein continues to mutate due to the change of the spike gene, angiotensin-converting enzyme 2 (ACE2), the binding to the major receptor, is impaired 157. These changes alter infection and mortality rates and reduce the efficacy of vaccines being developed based on the original COVID-19 strain. The effects and efficacy against the COVID-19 mutant using BNT162b2 (Pfizer/BioNTech) and mRNA-1273 (Moderna), mRNA-LNP vaccines that have passed FDA approval 157, are summarized in **Table 5**.

### Cancer LNP-based mRNA therapeutics vaccines and therapeutics

mRNA vaccines promote specific immune responses while encoding antigens which can be presented to antigen-presenting cells (APCs) 19. These antigens regulate the immune response against a tumor through two T-cell responses. One antigen presented by major histocompatibility complex (MHC) class 1 makes an immune response with CD8+ T cells. Another antigen released by lysosomes presented by MHC class 2 induces an immune response with CD4+ T cells 158, 159.

Cancer vaccines have a dual role, functioning as both a prophylactic and therapeutic. The mutations of tumor cells during carcinogenesis produce modified proteins called neoantigens. Utilizing neoantigens can produce customized/personalized neoepitopes for cancer vaccines to improve the anti-tumor immune responses compared to conventional cancer vaccines as shown in **Table 6** 160.

BNT111, IVAC MUTANOME (FixVAC), from BioNTech RNA Pharmaceuticals GmbH's is a poly neoantigen encoding RNA vaccine that targets unique mutational features in individual patients. From December 2013 to February 2017, a poly neoantigen immune response to the vaccine antigen was detected in all patients, and 60% of the 125 selected neonatal epitopes induced CD8+ and CD4+ T cell responses in malignant melanoma patients aged 18-49 years. (NCT02035956). Next, FixVac (BNT111), a Lipo-Mutanome Engineered RNA Immuno-Therapy (Lipo-MERIT), based on a nanoparticulate liposomal RNA (RNA-LPX) formulation, is a mRNA vaccine encoding four non-mutated antigens, also called tumor-associated antigens (TAAs), and has undergone a phase 1 clinical trial in patients with advanced melanoma. Most patients showed recruitment of the potent CD4+ and CD8+ T-cell immunity to antigens and a synergistic event with the combination of FixVac and anti-programmed death 1 (PD-1) in patients with experience with checkpoint inhibitors, demonstrating that TAAs may be useful as cancer vaccine targets (NCT02410733) 8. Based on a previous phase 1 clinical trial, the phase 2 clinical trial of BNT111 which in combination with Libtayo (Cemiplimab) in patients with unresectable stage 3 or 4 melanoma was started on May 19, 2021 (NCT04526899).

From October 2016 to May 2020, BioNTech SE's TNBC-MERIT was being conducted in 42 triple-negative breast cancer (TNBC) patients over the age of 18 years in a phase 1 clinical trial. The group treated with a combination of the IVAC_WAREHOUSE_bre1_uID and IVAC_MUTANOME_uID vaccine showed a strong induction of CD8+ T cells (NCT02316457) 108, 161.

Because neoantigens generated by somatic changes in cancer cells are very different between individual patients, it is necessary to develop a personalized vaccine. Moderna developed mRNA-4157, a mRNA encoding 43 neoantigens, and conducted phase 1 clinical trials on 142 solid cancer patients over the age of 18 years since August 2017. They divided patients with a resected solid cancer treated with mRNA alone and patients with an unresectable solid cancer or melanoma treated with pembrolizumab in combination into four groups. The median progression survival was longer in the group with the combination therapy (9.8 months) than in the group with the monotherapy (8 months) (NCT03313778). After entering the phase 2 clinical trial from January 2019, 150 patients aged 18 years and older were evaluated with mRNA-4157 and pembrolizumab as a postoperative adjuvant therapy that improves recurrence free survival compared to pembrolizumab only in patients with a high risk of recurrence of completely resected melanoma (NCT03897881). Similarly, in May 2018, there was also a first clinical trial using mRNA-4650, which encodes 20 neoantigens from 4 gastrointestinal cancer patients. Although a clinical response could not be obtained through a phase ½ clinical trial, it was able to elicit a response from CD4+ T cells (NCT03480152) 108, 162.

Instead of vaccination, clinical trials using mRNA-LNP drugs that encode co-stimulator molecules that enhance T-cell activity in the pro-inflammatory phase or deliver cytokines are also underway. mRNA-2416 is an OX40L (ligand of OX40)-encoding mRNA-LNP therapeutic. In August 2017, based on the results of the evaluation of antitumor efficacy in syngeneic colon and ovarian carcinoma tumor models in MC38-S and ID8 mice, a clinical phase ½ trial was conducted for the OX40L-encoding mRNA-LNP treatment of 117 patients over the age of 18 years with a solid tumor or lymphoma. This is a human first, phase 1, dose escalation (1 to 8 mg) clinical study, and the study was designed to determine the safety and efficacy of repeated intratumorally injections of mRNA-2416 alone and intravenous injection of a combination with durvalumab for patients. It was confirmed that the response of OX40L and T cells was increased at the injection site in the tumor (NCT03323398) 108.

As another mRNA-LNP treatment, mRNA-2752 encoding OX40L, pro-inflammatory cytokines, interleukin-23 (IL-23), and interleukin-36 gamma (IL-36γ) was injected 7 times for 2 weeks with dose escalation, followed by a combination therapy with durvalumab in 126 patients over the age of 18 years with a solid tumor or lymphoma. Both monotherapy and combination therapy of duravalumab have been shown to be associated with tumor shrinkage in lesions, and these data suggest sustained immunomodulatory effects of TNF-α, IFN-γ, and programmed death ligand 1 (PD-L1) with clinically low toxic levels in tumor and plasma assays (NCT03739931) 163.

Autogene cevemeran (RO7198457) is an mRNA vaccine designed to stimulate T-cell responses against neoantigens with iNeST's Lipo-MERIT. From December 2017 until now, the phase 1a / 1b clinical trial has been conducted with 25 to 100 ug of RO7198457 as a single therapy or in combination with 1200 mg of anti-PD-L1, Atezolizumab, being given to patients of melanoma, NSCLC, TNBC, colon cancer, and bladder cancer. In phase 1a, in the peripheral blood of 14 out of 16 patients (87%), T cell responses against RO7187457-induced neoantigens were observed by *ex vivo* ELISPOT or MHC multimer assays. In addition, it showed the activity of an innate immunity inducer because pro-inflammatory cytokines were released according to each dose. In the 1b clinical trial, 142 patients with various solid tumors were treated once every 3 weeks (Q3W) while increasing the dose of RO7198457 from 3 doses at 25 μg to 50 μg while fixing the dose of atezolizumab to 1200 mg. In TNBC patients, more than 5% of the CD8 T scale was induced in the peripheral blood, and there was mainly an effector memory phenotype and high expression of CD8+PD-1 of 99.2% (NCT03289962) 164.

## Utilization of *in vivo* imaging for development of LNP-based RNA therapeutics

The major challenge of *in vivo* delivery of RNA vaccines and therapeutics is that RNA molecules are highly unstable to physiological conditions due to ubiquitous RNases and are quickly cleared following systemic circulation. Therefore, LNPs are used as the RNA delivery system, protecting RNA from degradation and showing a longer circulation half-time than naked RNA 165. LNPs are also used to facilitate intracellular uptake (endocytosis), endosomal escape, and reduce undesired immunotoxicity 166, 167. Given the advantages of RNA delivery research using LNP, the PK and PD profiles are important biopharmaceutical aspects after LNP administration.

**Figure 6:**
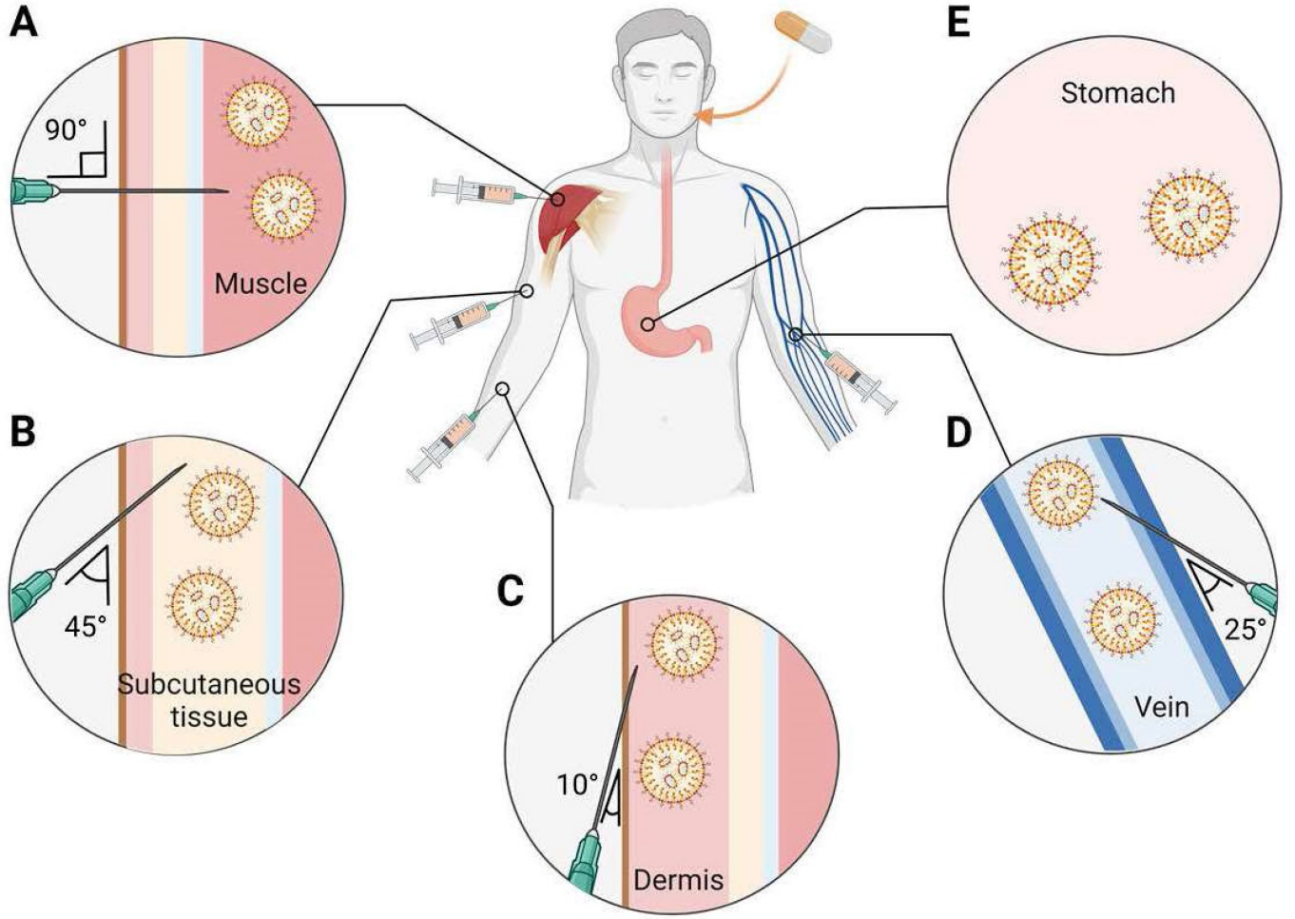
Main systemic delivery routes of RNA-LNPs. The selection of an appropriate route of administration may affect the efficacy of RNA medicines. RNA-LNPs are generally administered by systemic injections: **(A)** intramuscular (IM), **(B)** subcutaneous (SC), **(C)** intradermal (ID), **(D)** intravenous (IV), and **(E)** oral route. Created with BioRender.com.

**Figure 6A-E** provides an overview of the main routes for LNP-based therapeutics administration which are subcutaneous (SC), intramuscular (IM), intravenous (IV), intradermal (ID), and oral. LNPs are usually injected through a systemic route: enteral and parental routes 168. IM injection, a parental route, is especially used as an RNA vaccine for infectious diseases and cancer. Recently, COVID-19 mRNA-LNP vaccines from Moderna and Pfizer/BioNTech have also been intramuscularly administered into the deltoid muscle. In particular, both mRNA-LNP vaccines have more than a 90% vaccine efficacy when inoculated twice with IM injection at a 3-4 week interval 130, 132. Continuously producing proteins in muscle cells is an advantage in effective nucleic acid-based vaccines administered by IM injection because muscles have abundant blood vessels. It can recruit various immune cells to the injection site, facilitate protein expression, and trigger the immune system 169. Generally, an IV administered LNP is deposited in the liver primarily; therefore, liver targeted LNP-based RNA therapeutics would prefer the IV administration. For example, Patisiran, an FDA approved LNP-based siRNA therapeutics, is administered through the vein because its target is hepatocyte produced transthyretin amyloid 170. Additionally, IV injection is used to treat genetic disorders 5.

**Figure 7:**
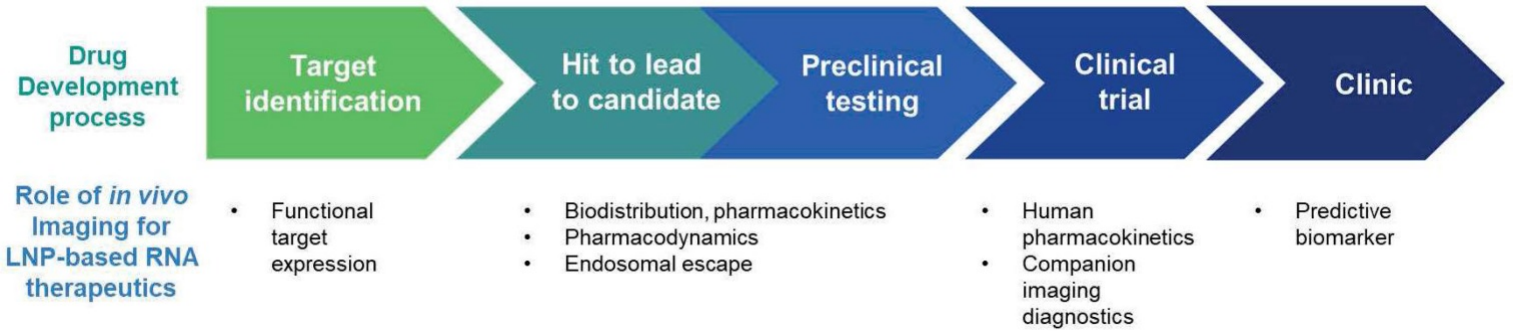
Potential role of imaging in development of LNP-based RNA therapeutics.

*In vivo* imaging has played an increasing role in the process of drug development. *In vivo* imaging has roles in accelerating the drug development process by the identification of drug targets, evaluation of the pharmacokinetics and pharmacodynamics of the drugs, and selection of optimal candidates 9, 10. Also, *in vivo* imaging can be used to develop companion imaging biomarkers which can facilitate patient selection for clinical trials. Furthermore, the imaging biomarkers that were developed for the clinical trials, can be utilized as predictive biomarkers in clinic as shown in **Figure 7** 9, 10, 171. Currently, *in vivo* imaging techniques is under-utilized in the process of development of LNP-based RNA Therapeutics. In this chapter, *in vivo* imaging techniques in the development of LNP-based RNA therapeutics will be summarized (**Table 7**) and describe areas that require future research.

### PD analysis using imaging LNP

**Figure 8:**
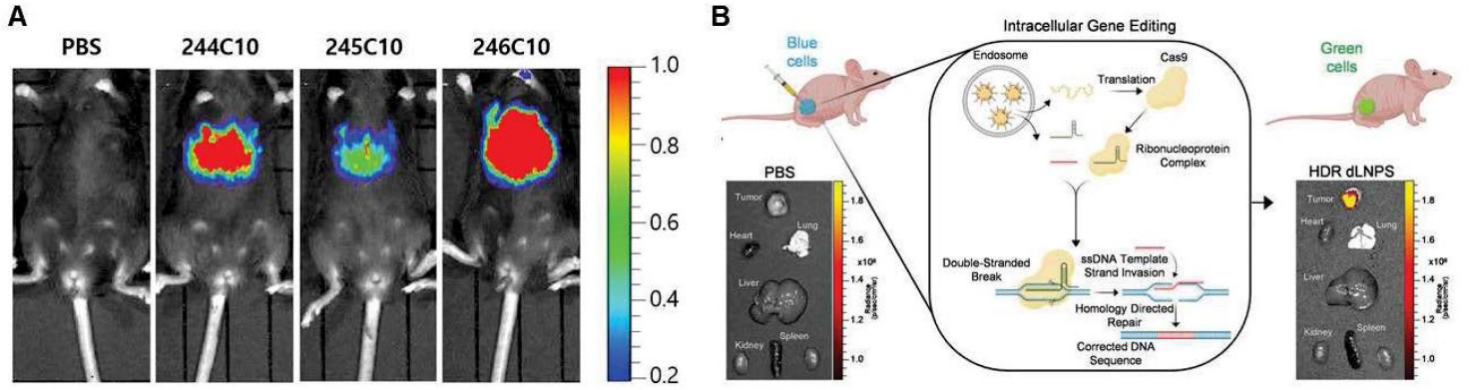
*In vivo* imaging of pharmacodynamics (PD) of LNP-based RNA therapeutics. **(A)** *In vivo* imaging was utilized to demonstrate luciferase gene expression, adapted from 174, and **(B)** gene editing efficacy, adapted from 176.

By the direct gene expression modulation ability of RNA therapeutics, PD can be easily evaluated by imaging. A fluorescent protein coding mRNA used for imaging can be used to assess the PD of the LNP for mRNA therapeutics. As a reporter gene formulated in LNPs, luciferase mRNA is effectively delivered to certain tissues or organs by avoiding metabolic enzymes by LNPs and is translated as a luminescent protein that can be visible using an IVIS (*In Vivo* Imaging System) 172, 173. Kim et al. utilized fluorescence *in vivo* imaging to show that different types of ionizable lipids have different *in vivo* efficacies in LNP formulations using LNPs loaded with luciferase genes 174 as shown in **Figure 8A**. *In vivo* imaging can elucidate different profiles of protein expression according to the administration routes. Pardi et al. compared the expression levels of firefly luciferase mRNA-LNPs administered at a dose of 0.005-0.250 mg/kg with six different injection routes [IV, intraperitoneal (IP), IM, SC, ID, and intratracheal (IT)]. The levels of protein translation were measured using *in vivo* imaging. Among the six groups, the IV administered groups showed the highest level of protein production. Meanwhile, the IM and ID injected groups showed the longest period (up to 10 days) of protein translation 21. Additionally, in the case of siRNA, gene silencing can be imaged *in vivo*. Tao et al. developed a mouse model with liver-specific expression of a luciferase gene, and a luciferase siRNA-loaded LNP was injected to assess the silencing effect of the siRNA therapeutics 175. In addition, genomic editing can be imaged using special cell lines. Farbiak et al. reported that dendrimer-based lipid nanoparticles (dLNPs) encapsulating three nucleic acids (Cas9 mRNA, sgRNA, and ssDNA) can edit the gene of BFP/GFP switchable HEK293 cells, and thus, the gene editing efficiency can be evaluated by fluorescence imaging *in vitro* and *in vivo* 176 shown in **Figure 8B**. *In vivo* imaging is a powerful tool to demonstrate the PD of LNP-based RNA therapeutics. However, they can elicit the pharmacological effect through multiple steps, which are delivered to the target tissue by endocytosis to the target cells, endosomal escape, and translation. Therefore, if the imaging showed a negative result, then it is hard to pinpoint which process was not efficient. In that sense, a separate evaluation of each step will facilitate the process of developing efficient LNP-based RNA therapeutics.

Moreover, for vaccines, the appropriate adaptive immune response is the most important aspect for their efficacy. mRNA-LNP vaccines can cause a prominent inflammatory response and induce a robust humoral response. LNPs can interact with pattern recognition receptors (PRRs) on antigen-presenting cells (APCs) and enhance pro-inflammatory cytokine release 177. Additionally, Ndeupen et al. reported that robust neutrophil infiltration was found in muscle tissue 24 hours after intramuscular injection of the LNP 178. This inflammatory response is considered as the causes for the side effect of vaccines and possibly reduce the efficacy to induce the adaptive immunity 179. Zhang et al. reported that an anti-inflammatory (dexamethasone incorporated) luciferase mRNA-loaded LNP showed improved luciferase protein expression compared to conventional LNPs 179. Therefore, careful assessment of the immune response by LNPs should be conducted during the development of LNP-based platforms 178, 180. Currently, tissue samples are used to evaluate the inflammatory process after administration of LNP-based platforms. However, there are various *in vivo* imaging modalities to non-invasively evaluate the inflammatory process 181. For example, ^18^F-fluorodexoyglucose PET can detect immune cells with increased metabolism, and is used clinically for the diagnosis of cardiac sarcoidosis and the differential diagnosis of fever of unknown origin (FUO) 182. Translocator protein (TSPO) tracers are also available for inflammation PET imaging. In particular, TSPO can be used for imaging neuroinflammation because TSPO is overexpressed in activated microglias 183. *In vivo* imaging has advantages in the assessment of the inflammatory process in the development of LNP-based RNA therapeutics because it is non-invasive and can be performed at multiple time points. Currently, inflammation imaging is not used for the development of LNP-based mRNA therapeutics, but it may facilitate the process of developing LNP-based mRNA therapeutics.

### PK analysis using *in vivo* imaging of LNP

*In vivo* imaging can be used for PK evaluation of LNPs. *In vivo* imaging can be done by labeling a contrast agent in the LNP or mRNA 174. The contrast agent can be iron oxide nanoparticles for magnetic resonance imaging, radionuclides for nuclear medicine imaging, an iodine agent for x-ray or CT imaging, or a fluorescence or luminescence dye for optical imaging. Fluorescent dyes are used most frequently in PK assessment of RNA therapeutics. Shi et al. reported that Cy5-labeled siRNA encapsulated LNP can be imaged *in vivo* to assess the biodistribution of the therapeutics. The siRNA showed high accumulation in the liver, spleen, and kidneys 184.

**Figure 9:**
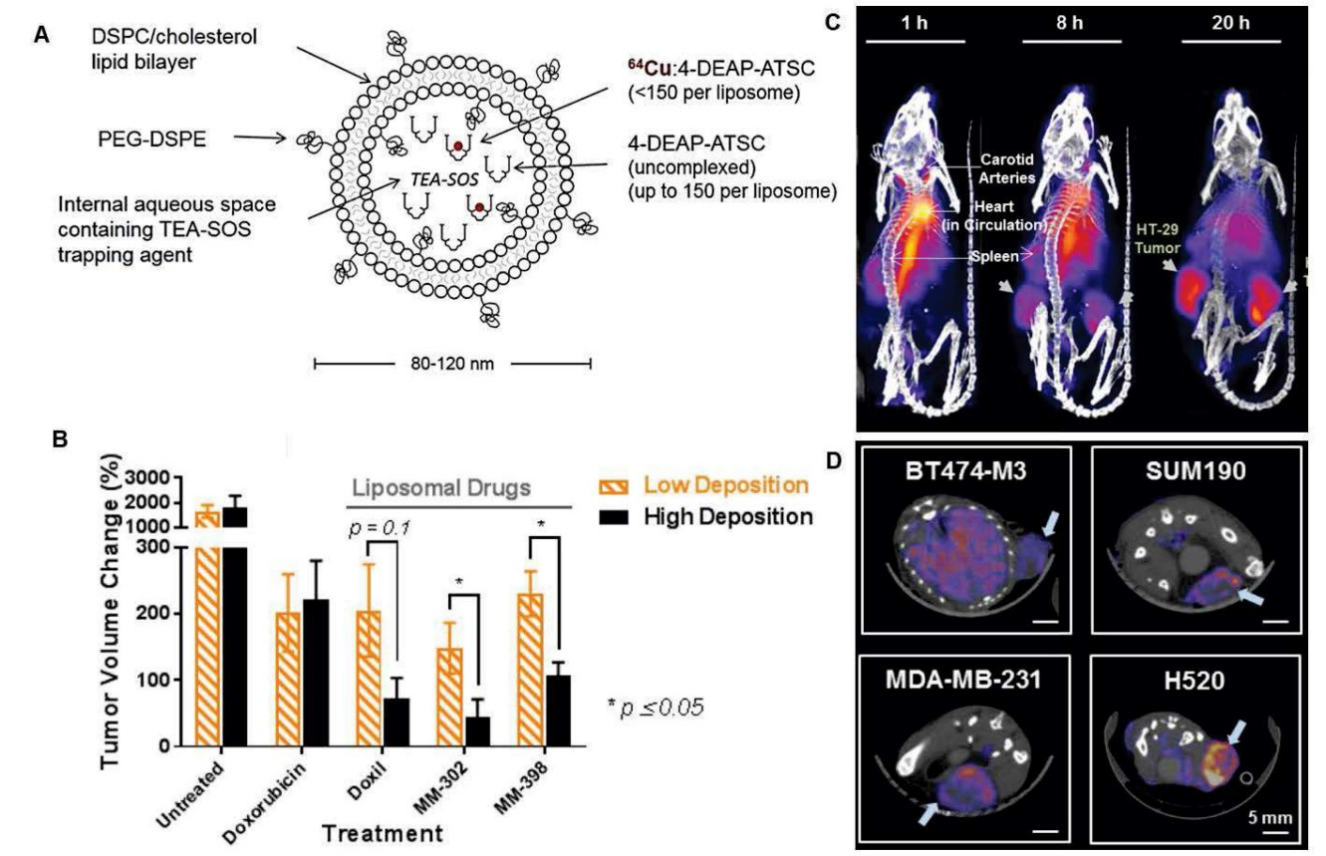
*In vivo* companion diagnostics using a radiolabeled liposome. **(A)** MM-DX-929 a companion diagnostic agent which is highly PEGylated untargeted liposome encapsulating ^66^Cu complexed with 4-DEAP-ATSC chelator. **(B)** Tumor volume change was significantly higher in tumor bearing mice with high deposition of MM-DX-929 compared to low deposition of the tracer. **(C)** Representative PET imaging of MM-DX-929 in tumor bearing mouse. **(D)** Axial PET/CT image shows different degrees of tumor MM-DX-929 uptakes, adapted from 188.

Among the various imaging methods, radionuclide imaging has the advantage to precisely assess the PK of drugs because of its high imaging sensitivity, and quantifiability 185. Although multiple studies have utilized radionuclide imaging techniques to evaluate the PK of LNP therapeutics encapsulating conventional agents, there are few papers that assess the PK of LNP encapsulated RNAs 186. Various radiolabeling methods of LNPs can be utilized to assess the PK of LNP-based RNA therapeutics 187. These methods include surface radiolabeling and intraliposomal labeling. For surface radiolabeling, radionuclides either with or without chelators can be attached to the membrane or incorporated into the lipid bilayer. On the other hand, radionuclides can be encapsulated within the hydrophilic core using ionophores, or radioactive compounds can be trapped after passive diffusion into the hydrophilic core 187. Radiolabeled LNPs can be used as a companion diagnostic for drug-loaded LNPs. Lee et al. developed a ^64^Cu labeled liposome for *in vivo* imaging companion diagnostics for another liposomal drug. They showed that a high accumulation of the ^64^Cu labeled liposome can predict a greater anti-tumor activity of other liposomal drugs shown in **Figure 9** 188. Although LNP encapsulating RNAs have a more complicated process to elicit a therapeutic effect than conventional liposomal drugs, the first step to achieve this goal will be the appropriate PK and that can be evaluated by *in vivo* imaging techniques. However, nucleic acid therapeutics can be damaged by ionizing radiation 189, and the integrity of RNA therapeutics using radionuclides for imaging should be analyzed before utilizing radionuclides in the PK evaluation of LNP-based RNA therapeutics.

### Endosomal escape analysis using the imaging of LNPs

**Figure 10:**
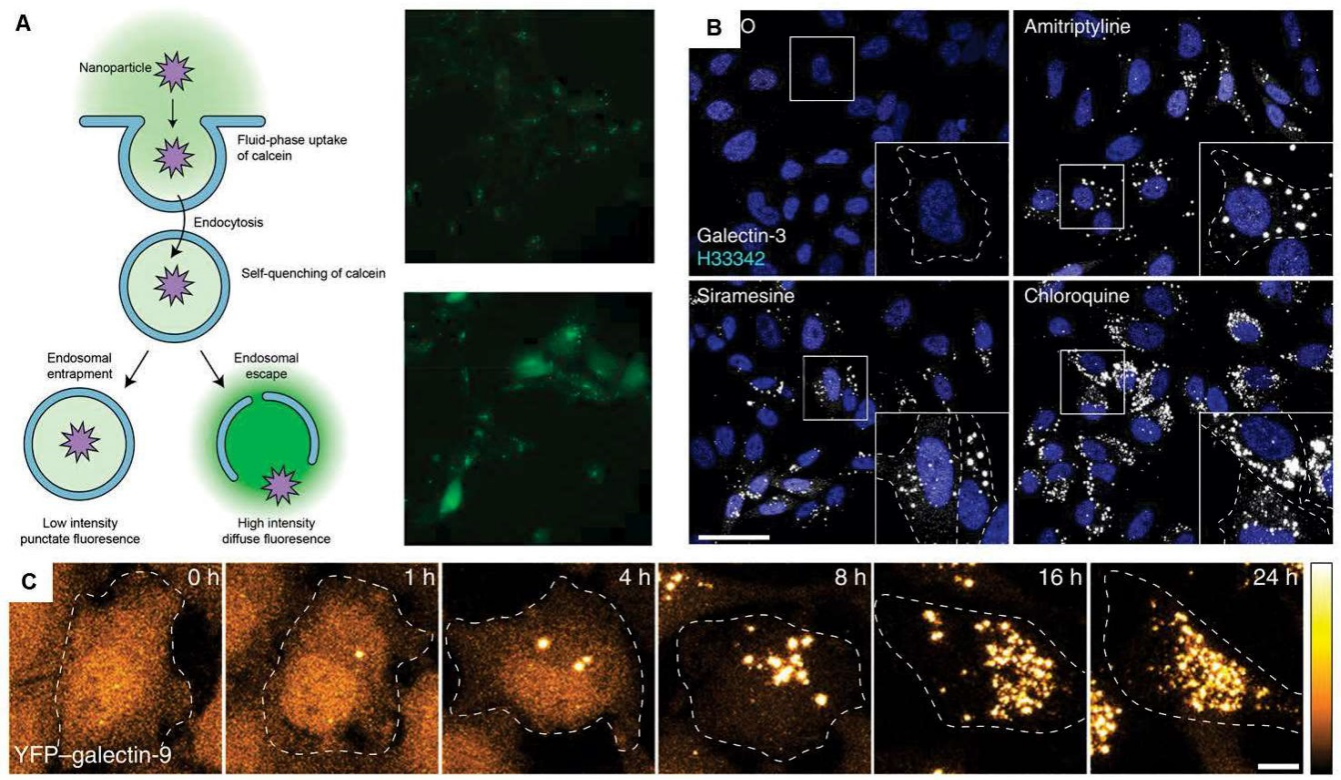
*In vitro* methods to assess the efficiency of endosomal escape. **(A)** Calcein leakage assay, adapted from 193. **(B)** Confocal images of HeLa cells stained for galectin-3 after treatment of various small molecules. **(C)** HeLa cells expressing YFP-galectin-9 after treatment with chloroquine, which can facilitate endosomal escape. Adapted from 196.

Endosomal escape is a crucial step for the efficiency of LNP-based RNA therapeutics. Nanoparticles including LNPs enter cells through endocytic pathway in most cases. Endocytic vesicles undergo unidirectional process which includes fusion with early endosomes, maturation into late endosomes, and fusion with lysosomes 190, 191. Lysosomes contain about 50 types of enzymes that can hydrolyze proteins, peptides, nucleic acids, lipids and polysaccharides 192. Therefore, LNP-based RNA therapeutics delivered to the cells should escape from endosomes to elicit its therapeutics effect. However, there is no established method to evaluate endosomal escape *in vivo*. Currently, there are several methods to detect *in vitro* endosomal escape. The methods are fluorescent labeling assays, leakage assays, membrane lysis assays, and transfection assays 193. The endosomal escape of fluorescent dye-labeled LNPs can be assessed by observation of the fluorescence signal pattern in the cells: a diffused pattern indicates endosomal escape while a punctate pattern implies the LNPs are still trapped 194. This method is not quantitative, and inter-rater agreement could be low. Leakage assays utilize small fluorescent molecules such as calcein. The fluorescence signal of calcein is quenched in endosomes/lysosomes due to the low pH and high local concentration of the dye, and when endosomal escape occurs, a diffuse fluorescence signal can be seen in the cytoplasm 195. Imaging membrane damage-associated markers can be done to evaluate endosomal escape. Rietz et al. reported that galectic-9 is a reliable marker for membrane damage and endosomal escape 196. However, this method can only be used in genetically engineered cells or in fixed cells shown in **Figure 10**. Because all currently reported studies evaluating endosomal escape are limited to *in vitro* applications, the development of *in vivo* methods to assess the endosomal escape of LNPs is required to facilitate the development of LNP-based RNA therapeutics.

## Conclusion

With the rapid and successful development of LNP-based mRNA vaccines against COVID-19, LNP-based mRNA therapeutics are considered one of the most promising therapeutic platforms. The PK and PD of drugs have a major impact on the efficacy and safety of the drugs. Currently, imaging of gene expression changes using fluorescent genes is frequently used to jointly evaluate the PK and PD of LNP-based mRNA therapeutics. However, because this method reflects the results of all the steps at once, it is difficult to use for RNA therapeutics to optimize each step to induce activity. *In vivo* imaging of LNP-based RNA therapeutics containing contrast agents could be utilized to optimize the biodistribution of the candidate LNP and to predict the therapeutic efficacy of the LNP. Endosomal escape is a crucial step for LNP-based RNA therapeutics, but an *in vivo* imaging method to evaluate endosomal escape is currently lacking. Therefore, it is warranted to develop appropriate *in vivo* imaging methods to evaluate the PK and endosomal escape to facilitate the successful development of LNP-based RNA therapeutics.

## Figures and Tables

**Figure 3:**
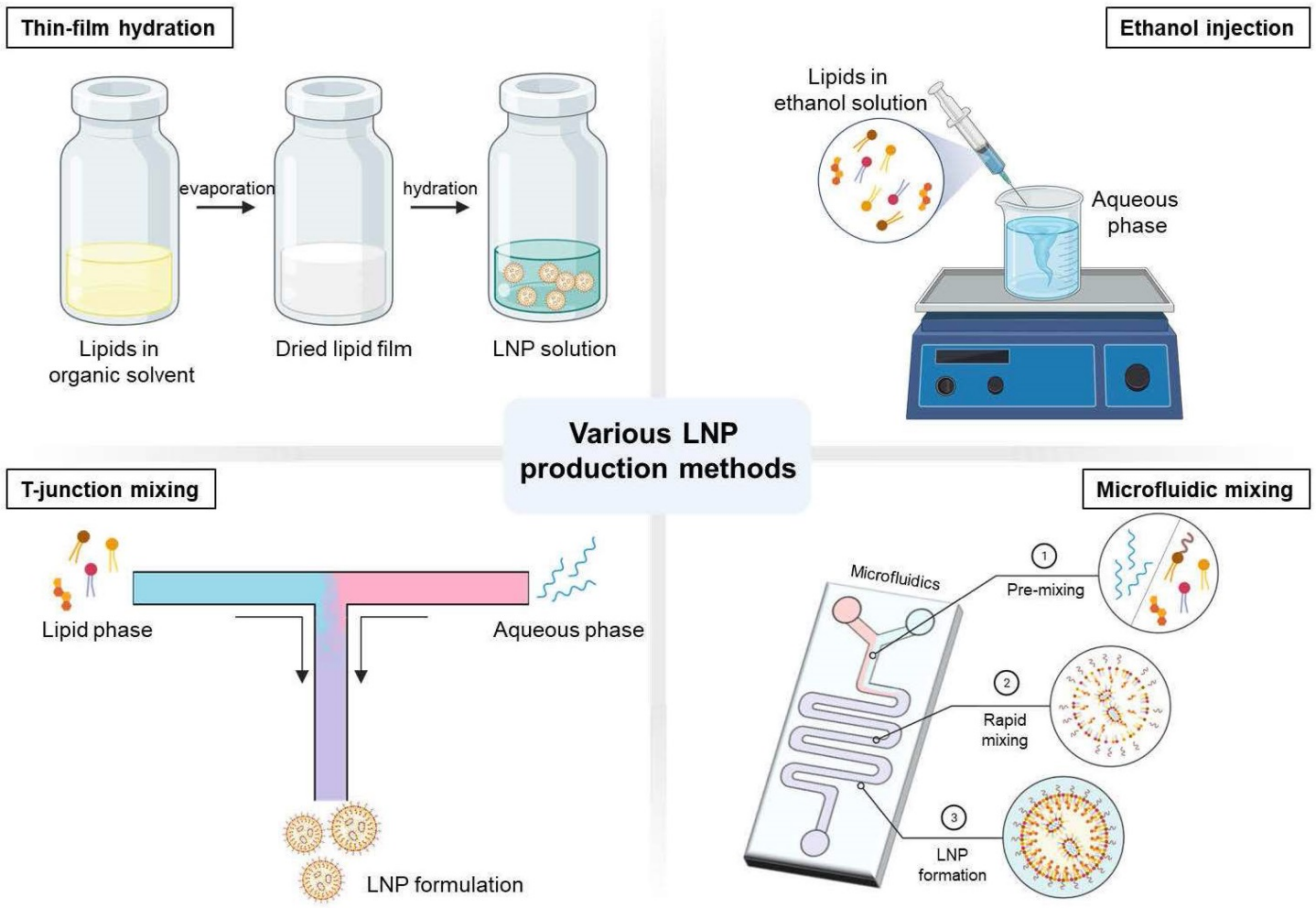
Illustration of various LNP production methods. Created with BioRender.com.

**Table 1:** LNP lipid components and their functions.

Lipid components	Functions	Examples
**Ionizable lipid**	- Nucleic acids complexation - Membrane fusion	- ALC-0315 (Pfizer/BioNTech) - SM-102 (Moderna)
**Phospholipid**	- Complex support - Provides highly stable structure (saturated lipids) and endosome destabilization (unsaturated lipids)	- DSPC, DPPC (saturated lipid) - DOPE (unsaturated lipid)
**Cholesterol**	- Integrity - Endosomal release	- Cholesterol
**PEGylated lipid**	- Hydrophilic surface - Steric hindrance - “Stealth” effect	- ALC-0159 (Pfizer/BioNTech) - PEG-DMG (Moderna)

**Table 2:** Advantages and disadvantages of each LNP production methods.

LNP Production Methods	Advantages	Disadvantages
**Thin-film hydration**	- Simple procedure - No expensive, complicated equipment needed	- Formulation of large multilamellar vesicles - Heterogeneous in size - Low encapsulation efficiency - Time consuming - Large-scale production difficulty - Difficult removal of organic solvent
**Ethanol injection**	- Simple procedure - Size controllable	- Time consuming - Low encapsulation efficiency - Difficult removal of organic solvent
**T-junction mixing**	- Reproducible & size controllable - Uniform particle formulation - High encapsulation efficiency - Large-scale production	- Difficult removal of organic solvent - Relatively high flow rate required - Lab-scale not preferred
**Microfluidic mixing**	- Reproducible & size controllable - Uniform particle formulation - High encapsulation efficiency - Easily scalable	- Difficult removal of organic solvent - Possible clogging in micro channel

**Table 3:** Representative clinical trials of delivery platforms with mRNA-LNPs against infectious diseases.

Name of vaccine / Company	Disease		Delivery platform	Encoding sequence	NCT number (ClinicalTrials.gov.identifier) / Phase
CV7201 / CureVAC AG	Rabies		RNAactive®	RABV-G	NCT02241135 / 1
CV7202 / CureVAC AG			mRNA-LNP	Glycoprotein G	NCT03713086 / 1
mRNA-1440 / Moderna	Influenza	H10N8	mRNA-LNP	Haemagglutinin	NCT03076385 / 1
mRNA-1851 / Moderna		H7N9			NCT03345043 / 1
mRNA-1893 / Moderna	Zika virus		mRNA-LNP	prM-E protein	NCT04064905 / 1
mRNA-1443 / Moderna	Cytomegalovirus		mRNA-LNP	pp65 T cell	NCT03382405 / 1
mRNA-1647 / Moderna				Pentamer complex and full-length membrane-attached glycoprotein B	NCT03382405 / 1 NCT04232280 / 2
mRNA-1273 / Moderna	SARS-CoV-2		mRNA-LNP	Full-length spike with Proline mutations (K986P, V987P, “2P”)	NCT04470427 / 3 (EMA) NCT05230953 / 3
BNT 162b2 / Pfizer-BioNTech					NCT04368728 / 3 (EMA) NCT05231005 / 3
CvnCoV / CureVAC AG					NCT04652102 / 2/3

EUA: Emergency Use Authorization; K: Lysine; P: Proline; prM-E: Pre-membrane and envelope; RABV-G: Rabies virus glycoprotein; SARS-CoV-2: Severe acute respiratory syndrome coronavirus 2.

**Table 4:** Characteristics of the EMA or FDA approved vaccines for COVID-19.

BNT162b2 (Pfizer / BioNTech)							
Lipid compositions	Lipid molar ratios	mRNA	Durability & storage	Administration	Efficacy	Adverse effect	Status
ALC-0315 (Ionizable lipid) ALC-0159 (PEG-DMA) DSPC (Helper lipid) Cholesterol (Helper lipid)	46.3:1.6: 9.4:42.7	Full-length spike with Proline mutations (K986P, V987P, “2P”) Nucleoside modified RNA (modRNA, N1-methylpseudoridine) Codon optimization (base 103-3879)	55 days (half-life) -80 ~ -60℃ (30 days), -25 ~ -15℃ (14 days)	Deltoid 30 μg / 0.3 mL	95% (90.0 ~ 97.9%, second dose with 21 days interval)	Fever Myocarditis Pericarditis	Ongoing Phase 3 trial (2020-2023, NCT04368728) FDA approval (August 23, 2021)
**mRNA-1273 (Moderna)**							
Lipid compositions	Lipid molar ratios	mRNA	Durability & storage	Administration	Efficacy	Adverse effect	Status
SM-102 (Ionizable lipid) PEG-DMG DSPC (Helper lipid) Cholesterol (Helper lipid)	50:1.5:10:5	Full-length spike with Proline mutations (K986P, V987P, “2P”) Nucleoside modified RNA (modRNA, N1-methylpseudoridine)	~55 days (half-life) 2 ~ 5℃ (30 days), -20 ~ -15℃ (4 months)	Deltoid 100 μg / 0.5 mL	94.1 % (89.3 ~ 96.8%, second dose with 28 days interval)	Fever Myocarditis Pericarditis	Completed Phase 3 trial (2020-2022, NCT04470427) FDA approval (January 31, 2022)

**Table 5:** Reported impact of the COVID-19 variants mRNA-LNP vaccine efficacy and effectiveness.

COVID-19 variant	Key mutations	Transmissibility	Vaccine-mediated protection	
			BNT162b2 (Pfizer/BioNTech)	mRNA-1273 (Moderna Therapeutics)
Reference strain	Reference strain	Reference strain	95%	94.1%
Alpha (B.1.1.7)	∆H69/V70, ∆Y144, N501Y, A570D, D614G, P681H	About 50% increase	90%	92%
Beta (B.1.351)	L18F, ∆L242, K417N, E484K, N501Y ,D614G, A701V	25% increase	75%	89%
Gamma (P.1)	L18F, E484K, K417N/T, N501Y, D614G	1.4 - 2.2 times more	82%	89%
Delta (B.1.617.2)	T95I, L452R, T478K, D614G, P681R	97% increase	88% (2 dose)	91% (2 dose)
Omicron (B.1.1.529)	G339D, S373P, S375F, K417N, N440K, S477N, T478K, E484A, Q493R, Q498R, N501Y, Y505H S371L, G446S, G496S (for BA.1) S371F, R408S (for BA.2)	~ 3.2 times (for Delta)	65.5% (2 dose) 67.2% (Booster)	75.1% (2 dose) 64.9% (Booster)

It may not be accurate due to the continuous updates in each country.

**Table 6:** Representative clinical trials of the delivery platforms with mRNA-LNPs against various cancers.

Name of vaccine / Sponsor	Disease	Delivery platform	Encoding sequence	ICI	ClinicalTrials.gov.identifier / Phase
FixVac (BNT111) / BioNTech RNA Pharmaceuticals GmbH	Melanoma	Lipo-MERIT	NY-ESO-1 Tyrosinase MAGE-A3 TPTE	Vemurafenib Dabrafenib Nivolumab Pembrolizumab Cemiplimab	NCT02410733 / 1 NCT04526899 / 2
BNT114 / BioNTech SE	TNBC	TNBC-MERIT	TAA	N.A.	NCT02316457 / 1
RO7198457 / BioNTech SE, Genentech	- NSCLC - Melanoma - TNBC - Colon cancer - Bladder cancer	Lipo-MERIT	Neoantigen	Atezolizumab	NCT03313778 / 1a, 1b NCT03897881 / 2
mRNA-4157 / Moderna Therapeutics	- NSCLC - Melanoma - Bladder urothelial cancer - HPV-HNSCC	mRNA-LNP	Neoantigen	Pembrolizumab	NCT03313778 / 1 NCT03897881 / 2
mRNA-4650 / Moderna Therapeutics	Metastatic melanoma	mRNA-LNP	Neoantigen	N.A.	NCT03480152 / 1
mRNA-2416 / Moderna Therapeutics	- Solid tumor - Lymphoma	mRNA-LNP	OX40L	Durvalumab	NCT03323398 / 1
mRNA-2752 / Moderna Therapeutics	- Solid tumor - Lymphoma	mRNA-LNP	OX40L, IL-23, IL-36y	Durvalumab	NCT03739931 / 1
mRNA-5671 / Moderna Therapeutics	- NSCLC - Colorectal cancer - Pancreatic adenocarcinoma	mRNA-LNP	KRAS mutations - G12D - G12V - G13D - G12C	Pembrolizumab	NCT03948763 / 1

C: Cysteine; D: Aspartate; G: Glycine; HPV-HNSCC: Human papillomavirus-negative head and neck squamous cell carcinoma; ICI: Immune checkpoint inhibitor; MAGE-3: Melanoma-associated antigen A3; MERIT: Mutanome Engineered RNA Immuno-Therapy; N.A.: Not applicable; NSCLC: Non-small cell lung cancer; NY-ESO-1: NewYork esophageal squamous cell carcinoma; OX40L: Ligand of OX40; TAA: Tumor associated antigen; TNBC: Triple negative breast cancer; TPTE: Transmembrane phosphatase with tensin homology; V: Valine.

**Table 7:** Current utilized imaging methods for evaluation of LNP-based RNA therapeutics.

	Imaging modality	Method	Interpretation	Limitation
Pharmacokinetics	Fluorescence imaging	- Fluorescence dye labeling to LNP or RNA	- *in vivo*: IVIS imaging - *in vitro*: Confocal microscopy	Not quantitative
Pharmacodynamics		- Fluorescent protein-coding mRNA - Silencing RNA for fluorescence genes (in cells or animals)		Assess multiple steps in one analysis
Endosomal escape		- Fluorescence labeling - Leakage assay - Membrane damage marker imaging	- *in vitro*: Confocal microscopy	Not quantitative and lacking *in vivo* imaging method
